# Adverse events with risankizumab in the real world: postmarketing pharmacovigilance assessment of the FDA adverse event reporting system

**DOI:** 10.3389/fimmu.2023.1169735

**Published:** 2023-05-15

**Authors:** Yamin Shu, Jing Chen, Yiling Ding, Qilin Zhang

**Affiliations:** ^1^Department of Pharmacy, Tongji Hospital, Tongji Medical College, Huazhong University of Science and Technology, Wuhan, China; ^2^Graduate School of Pharmaceutical Sciences, University of Tokyo, Tokyo, Japan; ^3^Department of Pharmacy, Union Hospital, Tongji Medical College, Huazhong University of Science and Technology, Wuhan, China

**Keywords:** adverse event, disproportionality, FAERS, pharmacovigilance, risankizumab

## Abstract

**Background:**

Risankizumab, a humanized IgG1 monoclonal antibody that selectively inhibits IL-23, is currently approved for the treatment of moderate-to-severe plaque psoriasis and Crohn’s disease. The real-world safety study of risankizumab in a large- sample population is currently lacking. The aim of this study was to evaluate risankizumab-associated adverse events (AEs) and characterize the clinical priority through the data mining of the Food and Drug Administration (FDA) Adverse Event Reporting System (FAERS).

**Methods:**

Disproportionality analyses were performed by calculating the reporting odds ratios (RORs), deemed significant when the lower limit of the 95% confidence interval was greater than 1, to quantify the signals of risankizumab-related AEs from the second quarter (Q2) of 2019 to 2022 Q3. Serious and non-serious cases were compared, and signals were prioritized using a rating scale.

**Results:**

Risankizumab was recorded in 10,235 reports, with 161 AEs associated with significant disproportionality. Of note, 37 PTs in at least 30 cases were classified as unexpected AEs, which were uncovered in the drug label, such as myocardial infarction, cataract, pancreatitis, diabetes mellitus, stress, and nephrolithiasis. 74.68%, 25.32%, and 0% PTs were graded as weak, moderate, and strong clinical priorities, respectively. A total of 48 risankizumab-related AEs such as pneumonia, cerebrovascular accident, cataract, loss of consciousness, cardiac disorder, hepatic cirrhosis, and thrombosis, were more likely to be reported as serious AEs. The median TTO of moderate and weak signals related to risankizumab was 115 (IQR 16.75–305) and 124 (IQR 29–301) days, respectively. All of the disproportionality signals had early failure type features, indicating that risankizumab-associated AEs gradually decreased over time.

**Conclusion:**

Our study found potential new AE signals and provided valuable evidence for clinicians to mitigate the risk of risankizumab-associated AEs based on an extensive analysis of a large-scale postmarketing international safety database.

## 1 Introduction

Psoriasis is a chronic, recurrent, immune-mediated inflammatory skin condition that affects approximately 3% of the US population and an estimated 125 million individuals globally (1, 2). Psoriasis affects men and women equally, but generally, adults are more affected than youngsters (3). Although psoriasis can present at any age, the age of onset of psoriasis is bimodal between the ages of 18–39 and 50–69 years (4). This may be due to the influence of genetic and environmental factors. Plaque psoriasis is the most common type of psoriasis, accounting for 90%. Psoriasis is characterized pathogenically by complex interactions between multiple immune cells, leading to aberrant immune responses and the upregulation of inflammatory cytokines, thus inducing excessive epidermal keratinocyte proliferation and the development of psoriasis plaques (5). Recently, biologic therapies have been frequently used as first-line treatment for moderate-to-severe plaque psoriasis, including tumor necrosis factor (TNF)–inhibitors, interleukin (IL)–12/23 monoclonal antibodies, IL-17A, and IL-23 inhibitors, which have shown promising efficacy in plaque psoriasis (6–8).

IL-23 is a key regulator of multiple effector cytokines, which has been implicated in the pathogenesis of psoriatic skin lesions, synovitis, and arthritis, making IL-23 inhibitors candidates for the treatment of plaque psoriasis and psoriatic arthritis (9, 10). Risankizumab, a humanized IgG1 monoclonal antibody that selectively binds to the p19 subunit of IL-23 and inhibits its interaction with the IL-23 receptor (IL-23R) complex, distinguishing it from biological therapies that target the shared IL-12 and IL-23 p40 subunit, is currently approved for the treatment of moderate-to-severe plaque psoriasis in over 70 countries, including the USA, Canada, and Japan and in Europe (11, 12). Subsequently, in June 2022, the FDA approved risankizumab for the treatment of Crohn’s disease based on the recent phase III trials, which revealed that risankizumab demonstrated excellent efficacy and safety as induction therapy in patients with moderate-to-severe Crohn’s disease who had not received or had received previous biologic therapy (13).

Although risankizumab is generally safe and well tolerated as other biological agents, they are associated with adverse effects possibly related to the mechanism of action, dose, or other factors. The long-term safety of risankizumab from 17 clinical trials have reported nasopharyngitis, upper respiratory tract infections, arthralgia, headache, and hypertension as the most common adverse events (AEs) (14). Serious AEs such as sepsis, pneumonia, cellulitis, breast cancer, and cardiovascular events are also observed.

As risankizumab is the latest IL-23 inhibitor and entered the market late, data on the safety of risankizumab in patients with chronic immune-mediated inflammatory diseases mainly come from clinical trials and postmarketing observational studies. The real-world safety study of risankizumab is currently lacking. In addition, real-world studies can make up for the shortcomings of clinical trials, such as strict inclusion and exclusion criteria and small sample sizes. Therefore, the aim of this study was to evaluate the long-term safety of risankizumab by postmarketing through the data mining of the FDA Adverse Event Reporting System (FAERS) database, which is a publicly accessible and the world’s largest pharmacovigilance database to detect AEs associated with drug exposure (15, 16).

## 2 Methods

### 2.1 Study design and data source

This retrospective, observational pharmacovigilance study used a disproportionality analysis to analyze whether an association is likely to exist between risankizumab and a given AE. Our study collected data from the FAERS Quarterly Data Extract Files between the second quarter (Q2) of 2019 (FDA marketing approval of risankizumab) and 2022 Q3 (the most recent update of the FAERS database at the time the study was performed). The FAERS database consists of seven orthogonal data files and an additional deleted file (16). Specific consent procedures were not required for this study because the FAERS database is publicly available. This study was approved (No. 20220185) by the institutional ethics board of the Union Hospital of Tongji Medical College of Huazhong University of Science and Technology.

### 2.2 Procedures

The data were downloaded from the FAERS website and then imported into MYSQL 8.0 for further processing. Due to the spontaneity and quarterly of the reports, duplicate reports are inevitable. We removed the duplicate reports according to the unique case ID, and the highest primary ID was reserved (17). Moreover, the deleted cases listed quarterly by the FDA for various reasons were also removed. We then extracted reports using the generic name (risankizumab-rzaa in drugname and prod_ai columns) and trade name (skyrizi in drugname column) in the DRUG file, which recorded drug/biologic information (18).

**Figure 1:**
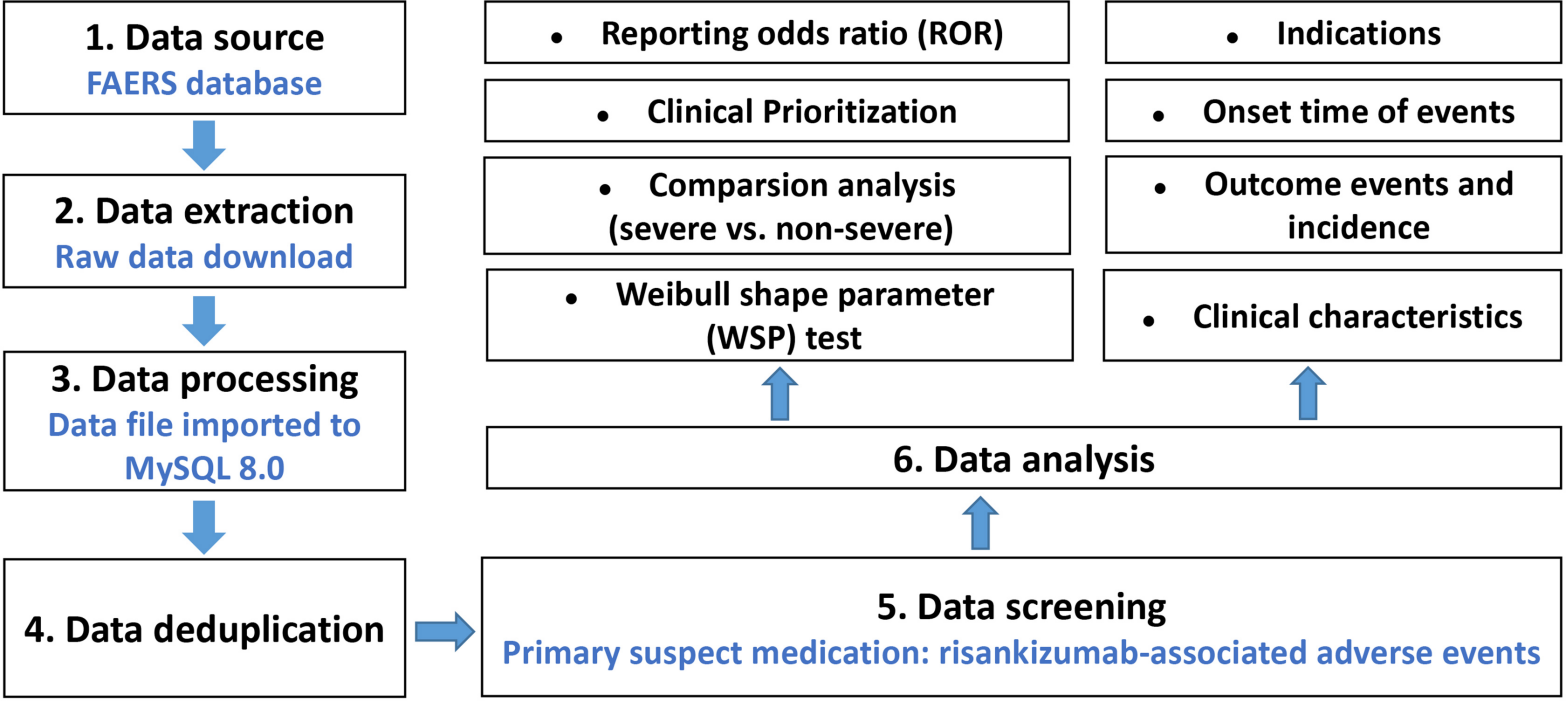
Multistep process of data extraction, processing, and analysis from the Food and Drug Administration adverse event reporting system database.

AEs submitted to the FAERS database were coded by standardized Medical Dictionary for Regulatory Activities (MedDRA). The preferred term (PT) and system organ class (SOC) in the structure of MedDRA 25.0 terminology were used in this study, and we performed case/non-case analysis to analyze if AEs were statistically significant reported with risankizumab as compared to other drugs in the full database at PT and SOC levels (15). When the data were available, the following clinical characteristics of reports were also calculated and analyzed: patient characteristics (gender, age, and weight), reporting countries, indications, outcomes (serious and non-serious), time-to-onset (TTO), concomitant medication, reporting year, etc. The detail of data identifying, extraction, and processing was shown in **Figure 1**. All data processing was performed using MYSQL 8.0 (Oracle, Sweden), Microsoft EXCEL 2019, and the GraphPad Prism 8 (GraphPad Software, CA, USA).

### 2.3 Statistical analysis

The reporting odds ratio (ROR), a disproportional signal detection method based on the principles of calculations using a 2 × 2 table, was employed to detect the association between AEs and risankizumab. Only the number of reports more than 10 were incorporated in our study. Generally, the higher the ROR value, the stronger the association between AEs and the target drug (19). A significant signal was detected when the ROR lower limit of the 95% confidence interval (CI) (ROR_025_) exceed 1 (20). The definition of 2 × 2 table and the calculation of ROR and ROR (95% two-sided CI) are shown in **Supplementary Table S1**. We listed serious and non-serious outcomes of reports separately, according to the report information in the FAERS, to clarify the severity of the detected significant signals and assess the potential risk factors (gender, age, and weight) in patients (21). Proportions were compared using Pearson’s chi-squared (χ^2^) or Fisher’s exact test, and the Mann–Whitney U test was applied for continuous non-normal distribution data, such as age and weight. Data were analyzed using SPSS (v22.0; IBM Corp., Armonk, NY, United States), and statistical significance was set at *P* < 0.05.

### 2.4 Clinical prioritization of signals

To identify reports of suspected AEs that deserve special attention in the framework of routine pharmacovigilance activities, irrespective of statistical criteria used to prioritize safety reviews, the designated medical event (DME) and important medical event (IME) list have been developed by the European Medicines Agency (EMA) (15, 22). Subsequently, a semiquantitative score method was created to rank the significant disproportionality AEs in terms of three levels of clinical importance by assessing five different features: the number of AE reports, ROR value, mortality proportion, IME or DME, and biological plausibility. The significant disproportionality AEs with weak, moderate, or strong clinical priority depend on the score between 0 and 4, 5 and 7, or 8 and 10, respectively. The detailed information is shown in **Supplementary Table S2**.

### 2.5 Time-to-onset analysis

TTO from the FAERS database was calculated from the start of risankizumab use to the occurrence of the AEs (23). The median (interquartile range, IQR), min–max, and the Weibull shape parameter (WSP) test were utilized in the evaluation of the TTO data for risankizumab (15, 24). We also performed the deduplication process and removed invalid data and only reports for which with TTO data were available for analysis. The WSP test is used to evaluate the characteristics of TTO data and can describe the non-constant rate of incidence of AEs (i.e., the risk of increase or decrease over time). The selection of the shape parameter and the differentiation criterion have been described in previous studies (25). The characteristics of TTO data, the WSP of signals with different prioritizations, and disproportional signals in the SOC level were calculated to describe the risk of occurrence of AE increase or decrease over time. All WSP test analyses were performed by Minitab statistical software (v20.0; Minitab LLC, State College, PA, United States).

## 3 Results

### 3.1 Descriptive analysis

During the study period, there were 5,754,056 AE reports in the FAERS, among which 10,235 (0.18%) cases were related to risankizumab (median [IQR] age, 59 (49–68) years). The detailed clinical characteristics are presented in **Table 1**. Gender data were available for 9,608 patients, and the proportion of women was higher than that of men (53.33% vs. 46.67%). Approximately 1,699 patients reported weight data, with the median weight of 87.17 kg. Risankizumab-associated AEs tended to occur in middle-aged patients (18–65 years, n = 3,009, 68.48%). The most common reported indication for risankizumab use was psoriasis (96.12%). Most cases came from the USA (n = 5,539, 83.72%). About 68.57% (n = 7,018) patients suffered from serious outcomes, including 2,507 hospitalizations, 531 death, and 69 disability with available follow-up. Additionally, 21.36% of the AE reports were submitted by healthcare professionals (n = 2,139), compared to 78.64% reported by consumers (n = 7,873). As shown in **Table 1**, there has been a marked increase in the number of AE reports with risankizumab from 2019 to 2022. Tozinameran, elasomeran, aspirin, metformin hydrochloride, and atorvastatin were the top five used drugs concomitantly for risankizumab-related AEs, with 756 (23.59%), 538 (16.79%), 224 (6.99%), 203 (6.33%), and 187 (5.83%) cases, respectively.

**Table 1:** Clinical characteristics of reports with risankizumab from the FAERS database (April 2019 to September 2022).

Characteristics	Risankizumab-induced AE reports (n = 10,235)		
Number of events	Available number, n	Case number, n	Case proportion, %
Gender, n (%)	9,608	–	93.87
Female	–	5,124	53.33
Male	–	4,484	46.67
Age (years), n (%)	4,394	–	42.93
<18	–	2	0.05
≤18 and ≤65	–	3,009	68.48
>65	–	1,383	31.47
Median (IQR)	–	59 (49–68)	–
Weight (kg), n (%)	1,699	–	16.60
<80	–	630	37.08
≤80 and ≤100	–	565	33.25
>100	–	504	29.66
Median (IQR)	–	87.17 (73.78–104.42)	–
Reported countries, n (%)	6,616	–	64.64
US	–	5,539	83.72
Non-US	–	1,077	16.28
Indications, n (%)	9,067	–	88.59
Psoriasis	–	8,715	96.12
Crohn’s disease		36	0.40
Outcomes, n (%)	10,235	–	100.00
Non-serious outcome	–	3,217	31.43
Serious outcome^a^	–	7,018	68.57
Death	–	531	7.57
Life-threatening	–	51	0.73
Hospitalization	–	2,507	35.72
Disability	–	69	0.98
Other serious outcomes	–	4,721	67.27
Time-to-onset (days)	2,498	–	24.41
Median (IQR)	–	114 (22–294.75)	–
Reporters, n (%)	10,012	–	97.82
Healthcare professionals	–	2,139	21.36
Consumer	–	7,873	78.64
Concomitant medication (TOP 5)	3,205	–	31.31
Tozinameran	–	756	23.59
Elasomeran	–	538	16.79
Aspirin	–	224	6.99
Metformin hydrochloride	–	203	6.33
Atorvastatin	–	187	5.83
Reporting year, n (%)	10,235	–	100.00
2022 Q3^b^	–	4,438	43.36
2021	–	3,871	37.82
2020	–	1,620	15.83
2019	–	306	2.99

a Total serious outcomes may exceed the total number of reported cases because some cases list more than one serious outcomes.

b The third quarter of 2022.

IQR, interquartile range. "-" means not applicable.

### 3.2 Disproportionality analysis

As described in **Table 2**, the disproportionality at the system organ class (SOC) level of risankizumab was observed in the FAERS. Risankizumab-induced AEs presented eight areas of toxicity with statistically significant RORs: skin and subcutaneous tissue disorders (n = 2,843, ROR 2.06, 95% CI: 1.98–2.15); infections and infestations (n = 2,038, ROR 1.88, 95% CI: 1.79–1.97); respiratory, thoracic, and mediastinal disorders (n = 1,828, ROR 1.26, 95% CI: 1.19–1.32); surgical and medical procedures (n = 1,697, ROR 5.45, 95% CI: 5.17–5.74); musculoskeletal and connective tissue disorders (n = 1,642, ROR 1.43, 95% CI: 1.36–1.51); immune system disorders (n = 1,606, ROR 1.69, 95% CI: 1.61–1.79); vascular disorders (n = 1,563, ROR 1.08, 95% CI: 1.02–1.14); and neoplasms benign, malignant, and unspecified (including cysts and polyps) (n = 1,391, ROR 1.26, 95% CI: 1.19–1.33).

**Table 2:** Signal strength of reports of risankizumab at the system organ class (SOC) level in the FAERS database.

System Organ Class (SOC)	Risankizumab cases reporting SOC	ROR (95% two-sided CI)
General disorders and administration site conditions	3,085	0.68 (0.66–0.71)
Skin and subcutaneous tissue disorders*	2,843	2.06 (1.98–2.15)
Infections and infestations*	2,038	1.88 (1.79–1.97)
Injury, poisoning, and procedural complications	1,948	0.56 (0.53–0.59)
Respiratory, thoracic, and mediastinal disorders*	1,828	1.26 (1.19–1.32)
Surgical and medical procedures*	1,697	5.45 (5.17–5.74)
Musculoskeletal and connective tissue disorders*	1,642	1.43 (1.36–1.51)
Nervous system disorders	1,629	0.76 (0.72–0.81)
Immune system disorders*	1,606	1.69 (1.61–1.79)
Vascular disorders*	1,563	1.08 (1.02–1.14)
Neoplasms benign, malignant, and unspecified (incl cysts and polyps)*	1,391	1.26 (1.19–1.33)
Cardiac disorders	1,127	1.03 (0.97–1.10)
Gastrointestinal disorders	1,096	0.53 (0.50–0.57)
Investigations	879	0.73 (0.68–0.78)
Psychiatric disorders	647	0.45 (0.41–0.48)
Renal and urinary disorders	534	0.68 (0.62–0.74)
Metabolism and nutrition disorders	423	0.58 (0.53–0.64)
Eye disorders	413	0.95 (0.86–1.05)
Reproductive system and breast disorders	412	0.72 (0.65–0.80)
Hepatobiliary disorders	268	0.92 (0.82–1.04)
Blood and lymphatic system disorders	251	0.46 (0.40-0.52)
Endocrine disorders	178	0.70 (0.60–0.81)
Product issues	150	0.31 (0.27–0.37)
Ear and labyrinth disorders	133	1.07 (0.90–1.26)
Social circumstances	105	0.81 (0.67–0.98)
Pregnancy, puerperium, and perinatal conditions	95	0.50 (0.41–0.61)
Congenital, familial, and genetic disorders	19	0.31 (0.20–0.49)

* indicates statistically significant signals in the algorithm.

ROR, reporting odds ratio; CI, confidence interval.

There are 161 different PTs of risankizumab-associated AEs reported in the FAERS in at least 10 cases. The number of reporting PTs ≥ 30 is listed in **Table 3**, including 79 PTs and 20 corresponding SOCs, and others are shown in **Supplementary Table S3**. Of note, among the 79 PTs, 37 PTs were classified as unexpected AEs in our data analysis, which were uncovered in the drug label, such as myocardial infarction, cataract, pancreatitis, diabetes mellitus, stress, nephrolithiasis, and thrombosis.

**Table 3:** Signal strength of the preferred term (PT) and the clinical priority assessing results (n ≥ 30).

SOC	PT	Cases (n)	ROR (95% two-sided CI)	Death (n)	IME/DME	Relevant evidence evaluation	Priority level (score)
Blood and lymphatic system disorders	Lymphadenopathy	30	1.90 (1.33-2.72)	1	NA	–	Weak (1)
Cardiac disorders	Myocardial infarction	205	4.59 (3.99-5.27)	20	IME	–	Weak (4)
	Atrial fibrillation	82	1.76 (1.42-2.19)	2	IME	–	Weak (3)
	Cardiac disorder	61	1.45 (1.13-1.87)	4	NA	++	Weak (4)
	Cardiac failure	56	1.50 (1.15-1.95)	6	IME	+	Weak (4)
	Coronary artery occlusion	55	14.44 (11.04-18.89)	1	IME	+	Moderate (6)
	Cardiac failure congestive	51	2.61 (1.98-3.43)	3	IME	+	Moderate (5)
Eye disorders	Cataract	171	6.15 (5.28-7.16)	0	IME	–	Moderate (5)
Gastrointestinal disorders	Pancreatitis	40	2.26 (1.66-3.09)	1	DME	–	Weak (4)
General disorders and administration site conditions	Therapeutic response shortened	127	7.46 (6.25-8.89)	0	NA	–	Weak (4)
	Unevaluable event	110	3.22 (2.67-3.89)	3	NA	–	Weak (3)
	Illness	100	1.64 (1.35-2.00)	4	NA	+	Weak (3)
	Hernia	59	6.22 (4.81-8.05)	0	NA	–	Weak (4)
	Injection site haemorrhage	49	1.42 (1.07-1.88)	0	NA	++	Weak (3)
	Impaired healing	37	2.8 (2.03-3.87)	1	NA	+	Weak (3)
	Gait inability	36	1.44 (1.04-2.00)	0	NA	–	Weak (1)
	Cyst	33	5.52 (3.91-7.78)	0	NA	–	Weak (3)
Hepatobiliary disorders	Hepatic cirrhosis	58	7.27 (5.60-9.42)	3	IME	+	Moderate (6)
	Cholelithiasis	48	4.52 (3.40-6.01)	0	NA	–	Weak (2)
	Liver disorder	33	1.72 (1.22-2.43)	1	NA	++	Weak (3)
Immune system disorders	Immunodeficiency	48	5.75 (4.32-7.64)	2	IME	+	Moderate (5)
Infections and infestations	COVID-19	564	3.88 (3.56-4.22)	33	NA	–	Weak (3)
	Pneumonia	248	1.64 (1.44-1.86)	16	IME	++	Moderate (5)
	Urinary tract infection	139	1.69 (1.43-2.00)	2	NA	++	Weak (4)
	Localized infection	69	5.53 (4.36-7.01)	0	NA	++	Moderate (6)
	Sepsis	68	1.33 (1.05-1.69)	4	IME	++	Moderate (5)
	Cellulitis	62	2.66 (2.07-3.42)	0	IME	++	Moderate (6)
	Tuberculosis	48	8.00 (6.01-10.64)	0	IME	++	Moderate (6)
	Diverticulitis	45	3.28 (2.44-4.39)	0	IME	–	Weak (3)
	Staphylococcal infection	40	3.11 (2.28-4.25)	0	NA	++	Weak (4)
	COVID-19 pneumonia	38	3.03 (2.20-4.16)	4	IME	–	Weak (3)
	Appendicitis	36	8.63 (6.21-12.01)	1	IME	–	Weak (4)
	Postprocedural infection	30	7.09 (4.94-10.17)	0	NA	++	Moderate (5)
Injury, poisoning, and procedural complications	Fall	325	2.16 (1.94-2.42)	12	NA	–	Weak (3)
	Maternal exposure during pregnancy	80	1.42 (1.14-1.78)	0	NA	–	Weak (2)
	Postprocedural complication	61	9.16 (7.11-11.80)	3	NA	++	Moderate (6)
	Procedural pain	54	4.57 (3.49-5.98)	0	NA	++	Moderate (5)
	Ankle fracture	48	6.59 (4.95-8.76)	0	NA	–	Weak (3)
	Road traffic accident	47	2.77 (2.08-3.69)	2	NA	–	Weak (2)
	Hip fracture	44	3.02 (2.24-4.06)	1	IME	–	Weak (3)
	Limb injury	41	2.75 (2.02-3.74)	0	NA	+	Weak (3)
	Upper limb fracture	37	3.71 (2.68-5.13)	1	NA	–	Weak (2)
	Accident	33	5.50 (3.90-7.75)	0	NA	–	Weak (3)
Investigations	SARS-CoV-2 test positive	86	6.02 (4.87-7.46)	2	NA	–	Weak (4)
	Oxygen saturation decreased	45	1.44 (1.08-1.93)	2	NA	–	Weak (1)
Metabolism and nutrition disorders	Diabetes mellitus	73	2.31 (1.83-2.91)	0	IME	–	Weak (4)
Musculoskeletal and connective tissue disorders	Pain in extremity	159	1.22 (1.04-1.43)	2	NA	++	Weak (4)
	Psoriatic arthropathy	78	3.52 (2.82-4.41)	0	IME	++	Moderate (6)
	Arthritis	75	1.91 (1.52-2.40)	0	NA	++	Weak (4)
	Mobility decreased	65	1.87 (1.47-2.39)	0	NA	–	Weak (2)
	Osteoarthritis	63	3.18 (2.48-4.07)	0	NA	++	Moderate (5)
	Arthropathy	52	1.69 (1.29-2.22)	0	NA	++	Weak (4)
	Musculoskeletal disorder	41	4.46 (3.28-6.07)	1	NA	++	Weak (4)
	Intervertebral disc protrusion	39	4.23 (3.08-5.80)	0	NA	–	Weak (2)
	Systemic lupus erythematosus	35	1.82 (1.30-2.54)	0	IME	–	Weak (2)
Nervous system disorders	Cerebrovascular accident	210	3.56 (3.11-4.09)	10	IME	+	Moderate (5)
	Loss of consciousness	108	2.08 (1.72-2.52)	4	IME	+	Moderate (5)
	Burning sensation	42	1.42 (1.05-1.92)	0	NA	++	Weak (3)
	Carpal tunnel syndrome	37	5.67 (4.10-7.85)	0	NA	–	Weak (3)
	Transient ischemic attack	31	2.88 (2.02-4.10)	0	IME	+	Weak (4)
Pregnancy, puerperium, and perinatal conditions	Abortion spontaneous	40	2.26 (1.66-3.09)	0	IME	–	Weak (3)
Product issues	Device issue	80	1.37 (1.10-1.71)	0	NA	++	Weak (4)
Psychiatric disorders	Stress	87	2.48 (2.00-3.06)	1	NA	–	Weak (3)
Renal and urinary disorders	Nephrolithiasis	80	3.49 (2.80-4.36)	0	IME	–	Weak (4)
Respiratory, thoracic, and mediastinal disorders	Chronic obstructive pulmonary disease	67	2.98 (2.34-3.79)	3	NA	–	Weak (3)
	Respiratory disorder	41	3.01 (2.21-4.10)	1	NA	++	Weak (4)
	Lung disorder	39	1.66 (1.21-2.28)	1	NA	++	Weak (3)
	Pulmonary edema	38	1.93 (1.41-2.66)	3	IME	–	Weak (2)
Skin and subcutaneous tissue disorders	Psoriasis	1058	17.99 (16.87-19.19)	1	NA	++	Moderate (6)
	Pruritus	269	1.54 (1.37-1.74)	0	NA	++	Weak (4)
	Erythema	152	1.24 (1.06-1.45)	1	NA	++	Weak (4)
	Skin disorder	117	7.74 (6.44-9.29)	1	NA	++	Moderate (6)
	Skin exfoliation	108	1.99 (1.64-2.40)	0	NA	+	Weak (3)
	Skin plaque	56	11.32 (8.69-14.76)	0	NA	+	Moderate (5)
	Skin discoloration	55	2.39 (1.83-3.11)	0	NA	+	Weak (4)
	Blister	42	1.61 (1.19-2.18)	1	NA	–	Weak (1)
	Skin fissures	34	3.53 (2.52-4.95)	0	NA	+	Weak (3)
Vascular disorders	Thrombosis	54	1.36 (1.04-1.78)	0	IME	–	Weak (3)
	Arterial occlusive disease	35	12.60 (9.01-17.62)	0	NA	–	Weak (3)

ROR, reporting odds ratio; CI, confidence interval; IME, important medical event; DME, designated medical event; NA, not applicable (for relevant criteria); n, number of cases. ++ : AEs are mainly from the FDA Prescribing Information, the Summary of Product Characteristics of risankizumab posted by the MHRA, Phase 2/3 RCTs, or systematic reviews, with biological plausibility. + : AEs are mainly from other clinical trials, observational studies, or case reports/series with potential biological plausibility. - : AEs only emerging from disproportionality analyses.

### 3.3 Clinical prioritization of relevant disproportionality signals

As shown in **Table 3**, in total, 26 out of the 79 PTs (32.91%) with statistically significant disproportionality signals were categorized as IMEs, and only 1 represented DMEs, including pancreatitis with an ROR of 2.26 (95% CI: 1.66–3.09). Based on the clinical priority score, 59 (74.68%), 20 (25.32%), and 0 PTs were graded as weak, moderate, and strong clinical priorities, respectively. Nine detected disproportionality signals were presented as moderate clinical priority with the highest priority score of 6 (i.e., coronary artery occlusion, hepatic cirrhosis, localized infection, cellulitis, tuberculosis, postprocedural complication, psoriatic arthropathy, psoriasis, and skin disorder). In the assessment of the relevant evidence, 27 PTs showed a strong level of evidence with “++.”

### 3.4 Serious vs. non-serious cases

In our data analysis, women accounted for a larger proportion than men (3,535 vs. 3,310). Furthermore, gender (χ^2 = ^27.218, *P* < 0.001) and age (60 vs. 57 years, *P* < 0.001) showed statistically significant differences between severe and non-severe cases of risankizumab-associated AEs (**Table 4**). On the contrary, weight did not differ between the two groups (86.26 vs. 88.53 kg, respectively, *P* = 0.082). In total, 48 risankizumab-related AEs in at least 30 cases, such as pneumonia, cerebrovascular accident, cataract, loss of consciousness, chronic obstructive pulmonary disease, cardiac disorder, hepatic cirrhosis, and thrombosis, were more likely to be reported as serious AEs with *P* < 0.05. It was noteworthy that all outcomes for AEs of hip fracture (n = 44), pulmonary fibrosis (n = 17), pulmonary mass (n = 17), thyroid disorder (n = 13), urosepsis (n = 13), precancerous cells present (n = 12), paternal exposure during pregnancy (n = 11), and poor peripheral circulation (n = 11) were severe cases (**Table 4** and **Supplementary Table S4**).

**Table 4:** Differences in clinical characteristics of serious and non-serious reports (n ≥ 30).

	Serious cases (n = 7,018)	Non-serious cases (n = 3,217)	Statistic	p-value
Gender, n (%)	–	–	–	–
Female	3,535 (68.99)	1589 (31.01)	27.218^b^	<0.001^a^
Male	3,310 (73.82)	1174 (26.18)		
Age, years (median, IQR)	60 (50-68)	57 (45-67)	-5.791^d^	<0.001^c^
Weight, kg (median, IQR)	86.26 (74-103.97)	88.53 (72.64-108.86)	-1.737^d^	0.082^c^
Types of AEs, n ≥ 30 (%)	–	–		
Psoriasis	626 (8.92)	432 (13.43)	48.381^b^	<0.001^a^
COVID-19	401 (5.71)	163 (5.07)	1.774^b^	0.183^a^
Fall	287 (4.09)	38 (1.18)	60.682^b^	<0.001^a^
Pruritus	149 (2.12)	120 (3.73)	22.261^b^	<0.001^a^
Pneumonia	193 (2.75)	55 (1.71)	10.099^b^	0.001^a^
Cerebrovascular accident	185 (2.64)	25 (0.78)	37.930^b^	<0.001^a^
Myocardial infarction	177 (2.52)	28 (0.87)	30.659^b^	<0.001^a^
Cataract	149 (2.12)	22 (0.68)	27.813^b^	<0.001^a^
Pain in extremity	112 (1.60)	47 (1.46)	0.263^b^	0.608^a^
Erythema	98 (1.40)	54 (1.68)	1.200^b^	0.273^a^
Urinary tract infection	98 (1.40)	41 (1.27)	0.245^b^	0.621^a^
Therapeutic response shortened	58 (0.83)	69 (2.14)	31.288^b^	<0.001^a^
Skin disorder	73 (1.04)	44 (1.37)	2.094^b^	0.148^a^
Unevaluable event	75 (1.07)	35 (1.09)	0.008^b^	0.930^a^
Loss of consciousness	101 (1.44)	7 (0.22)	31.527^b^	<0.001^a^
Skin exfoliation	76 (1.08)	32 (0.99)	0.164^b^	0.685^a^
Illness	73 (1.04)	27 (0.84)	0.920^b^	0.337^a^
Stress	63 (0.90)	24 (0.75)	0.602^b^	0.438^a^
SARS-CoV-2 test positive	44 (0.63)	42 (1.31)	12.192^b^	<0.001^a^
Atrial fibrillation	71 (1.01)	11 (0.34)	12.450^b^	<0.001^a^
Device issue	12 (0.17)	68 (2.11)	107.357^b^	<0.001^a^
Maternal exposure during pregnancy	52 (0.74)	28 (0.87)	0.476^b^	0.490^a^
Nephrolithiasis	71 (1.01)	9 (0.28)	15.237^b^	<0.001^a^
Psoriatic arthropathy	44 (0.63)	34 (1.06)	5.391^b^	0.020^a^
Arthritis	49 (0.70)	26 (0.81)	0.367^b^	0.545^a^
Diabetes mellitus	52 (0.74)	21 (0.65)	0.242^b^	0.623^a^
Localized infection	56 (0.80)	13 (0.40)	5.110^b^	0.024^a^
Sepsis	58 (0.83)	10 (0.31)	8.885^b^	0.003^a^
Chronic obstructive pulmonary disease	60 (0.85)	7 (0.22)	13.778^b^	<0.001^a^
Mobility decreased	49 (0.70)	16 (0.50)	1.410^b^	0.235^a^
Osteoarthritis	48 (0.68)	15 (0.47)	1.709^b^	0.191^a^
Cellulitis	43 (0.61)	19 (0.59)	0.018^b^	0.894^a^
Cardiac disorder	51 (0.73)	10 (0.31)	6.439^b^	0.011^a^
Postprocedural complication	50 (0.71)	11 (0.34)	5.112^b^	0.024^a^
Hernia	50 (0.71)	9 (0.28)	7.206^b^	0.007^a^
Hepatic cirrhosis	52 (0.74)	6 (0.19)	12.034^b^	0.001^a^
Cardiac failure	50 (0.71)	6 (0.19)	11.213^b^	0.001^a^
Skin plaque	34 (0.48)	22 (0.68)	1.612^b^	0.204^a^
Coronary artery occlusion	48 (0.68)	7 (0.22)	8.976^b^	0.003^a^
Skin discoloration	40 (0.57)	15 (0.47)	0.444^b^	0.505^a^
Procedural pain	47 (0.67)	7 (0.22)	8.591^b^	0.003^a^
Thrombosis	48 (0.68)	6 (0.19)	10.401^b^	0.001^a^
Arthropathy	43 (0.61)	9 (0.28)	4.838^b^	0.028^a^
Cardiac failure congestive	43 (0.61)	8 (0.25)	5.896^b^	0.015^a^
Injection site hemorrhage	6 (0.09)	43 (1.34)	72.473^b^	<0.001^a^
Ankle fracture	38 (0.54)	10 (0.31)	2.513^b^	0.113^a^
Cholelithiasis	43 (0.61)	5 (0.16)	9.882^b^	0.002^a^
Immunodeficiency	34 (0.48)	14 (0.44)	0.115^b^	0.735^a^
Tuberculosis	43 (0.61)	5 (0.16)	9.882^b^	0.002^a^
Road traffic accident	37 (0.53)	10 (0.31)	2.259^b^	0.133^a^
Diverticulitis	39 (0.56)	6 (0.19)	6.869^b^	0.009^a^
Oxygen saturation decreased	40 (0.57)	5 (0.16)	8.660^b^	0.003^a^
Hip fracture	44 (0.63)	0 (0.00)	20.256^b^	<0.001^a^
Blister	27 (0.38)	15 (0.47)	0.359^b^	0.549^a^
Burning sensation	22 (0.31)	20 (0.62)	5.128^b^	0.024^a^
Limb injury	33 (0.47)	8 (0.25)	2.714^b^	0.100^a^
Musculoskeletal disorder	35 (0.50)	6 (0.19)	5.389^b^	0.020^a^
Respiratory disorder	33 (0.47)	8 (0.25)	2.714^b^	0.100^a^
Abortion spontaneous	39 (0.56)	1 (0.03)	15.596^b^	<0.001^a^
Pancreatitis	35 (0.50)	5 (0.16)	6.678^b^	0.010^a^
Staphylococcal infection	35 (0.50)	5 (0.16)	6.678^b^	0.010^a^
Intervertebral disc protrusion	34 (0.48)	5 (0.16)	6.292^b^	0.012^a^
Lung disorder	32 (0.46)	7 (0.22)	3.302^b^	0.069^a^
COVID-19 pneumonia	33 (0.47)	5 (0.16)	5.910^b^	0.015^a^
Pulmonary edema	33 (0.47)	5 (0.16)	5.910^b^	0.015^a^
Carpal tunnel syndrome	31 (0.44)	6 (0.19)	3.989^b^	0.046^a^
Impaired healing	30 (0.43)	7 (0.22)	2.698^b^	0.101^a^
Upper limb fracture	31 (0.44)	6 (0.19)	3.989^b^	0.046^a^
Appendicitis	34 (0.48)	2 (0.06)	11.224^b^	0.001^a^
Gait inability	26 (0.37)	10 (0.31)	0.224^b^	0.636^a^
Arterial occlusive disease	27 (0.38)	8 (0.25)	1.198^b^	0.274^a^
Systemic lupus erythematosus	31 (0.44)	4 (0.12)	6.520^b^	0.011^a^
Skin fissures	21 (0.30)	13 (0.40)	0.733^b^	0.392^a^
Accident	28 (0.40)	5 (0.16)	4.071^b^	0.044^a^
Cyst	28 (0.40)	5 (0.16)	4.071^b^	0.044^a^
Liver disorder	27 (0.38)	6 (0.19)	2.697^b^	0.101^a^
Transient ischemic attack	25 (0.36)	6 (0.19)	2.104^b^	0.147^a^
Lymphadenopathy	21 (0.30)	9 (0.28)	0.029^b^	0.866^a^
Postprocedural infection	26 (0.37)	4 (0.12)	4.573^b^	0.032^a^

The AEs listed above were AEs with significant signal strengths (n ≥ 30).

a Proportions were compared using the Pearson χ^2^ test.

b The χ^2^ statistic of the Pearson chi-square test.

c Mann–Whitney U test.

d The Z statistic of the Mann–Whitney U test.

*p*-values less than 0.05 were considered statistically significant.

### 3.5 Time-to-onset analysis

Results of TTO and WSP analyses for signals with different prioritizations are shown in **Table 5**. The median TTO of moderate and weak signals related to risankizumab was 115 (IQR 16.75–305) and 124 (IQR 29–301) days, respectively. According to the WSP analysis, both the shape parameter β and their 95% CI upper limit were < 1, revealing that an early failure type existed in these clinical priority signals.

**Table 5:** The results of time-to-onset analysis for signals with different prioritizations.

Prioritization	TTO (days)			Weibull distribution				Failure type
	Cases			Scale parameter		Shape parameter		
	n	Median (IQR)	Min–max	α	95% CI	β	95% CI	
Moderate	680	115 (16.75–305)	0–962	133.25	115.07–154.29	0.53	0.49–0.56	Early failure
Weak	1,744	124 (29–301)	0–1096	145.18	133.34–158.08	0.57	0.55–0.59	Early failure

n, number of cases with available time-to-onset; IQR, interquartile range; TTO, time-to-onset. When TTO is 0 days, the adverse event occurred within the same day with the therapy.

The results of TTO analysis for signals in different SOC levels were presented in **Supplementary Table S5**. Reproductive system and breast disorders, neoplasms benign, malignant and unspecified (incl cysts and polyps), metabolism and nutrition disorders, and blood and lymphatic system disorders represented the longest median TTO with 202 (IQR 34–351.25), 193 (IQR 62–351), 188 (IQR 32–371), and 180 (IQR 54.5–334.5) days, while ear and labyrinth disorders and skin and subcutaneous tissue disorders had the shortest median TTO with 29.5 (IQR 10–190) and 78 (IQR 14–266) days, respectively.

## 4 Discussion

To the best of our knowledge, previous safety studies of risankizumab were limited to clinical trials or concentrated on specific AEs, such as cerebrovascular accident, which might provide only a narrow opinion of potential safety issues (13, 26–28). In this first large real-world pharmacovigilance study, it summarized the worldwide reporting of risankizumab-associated AEs in the FAERS most comprehensively and systematically. Additionally, the clinical priority score was innovated and used to assign clinical relevance to signals, thus avoiding unnecessary alarms.

In view of the increased number of approved indications and widespread use of risankizumab, the associated AEs significantly increased from 2019 to 2022, with the annual reports in 2021 (n = 3,871) almost 12 times of 2019 (n = 306). It was reported that psoriasis always occurred in young adults, and up to 30% of patients presented with their first symptoms during childhood and adolescence (3). Moreover, it had two main peaks of onset at approximately 20–30 and 50–60 years of age (3), which was basically consistent with the current study that the primary age of onset of reported AEs induced by risankizumab was in 18–65 years (68.48%). However, there is no obvious difference in morbidities between genders (29, 30). In our study, the proportion of women was slightly higher than that of men (53.33% vs. 46.67%). Moreover, sex (*P* < 0.001) and patient age (*P* < 0.001) rather than body weight (*P* = 0.082) might be associated with an increased risk of risankizumab-induced AE severity when compared between serious cases and non-serious cases (**Table 4**).

Our disproportionality analysis showed that the most common and significant SOCs of risankizumab were focused on infections and infestations; skin and subcutaneous tissue disorders; respiratory, thoracic, and mediastinal disorders; musculoskeletal and connective tissue disorders; vascular disorders, and neoplasms, which were in line with the safety data in the label and clinical trials (4). Among the SOC of infections and infestations, the most reported AEs associated with risankizumab were COVID-19 (n = 564), pneumonia (n = 248), and urinary tract infection (n = 139). Previously, several clinical trials reported that the most common AEs of risankizumab were worsening Crohn’s disease, nasopharyngitis, arthralgia, headache, nausea, abdominal pain, diarrhea, and anemia (13, 31, 32). Similar to risankizumab, these common AEs were observed in plaque psoriasis patients assigned to active treatment arms in clinical trials of other IL-23 inhibitors, IL-17A inhibitors, and TNF inhibitors (16, 30, 33). However, our study differed in that the most frequently reported AEs were COVID-19, pneumonia, pruritus, cerebrovascular accident, and myocardial infarction. Furthermore, these AEs might result in serious outcomes with evidence in the present study that statistically significant differences were observed between the severe and non-severe cases (*P* < 0.001). This is consistent with a study conducted by Mease et al., in which serious infections were reported in two patients who received risankizumab. One of the patients reported severe urinary tract infection, influenza, and sepsis and had a severe malignancy (stage IV ovarian cancer) and three major adverse cardiovascular events (MACEs) (acute myocardial infarction, congestive heart failure, and coronary artery occlusion) (27).

Cardiovascular and cerebrovascular safety has become a concern after some clinical trials that observed an increased incidence of MACE and cerebrovascular accident with psoriasis biologics (34, 35). In the present study, although we observed significant AE signals of myocardial infarction (ROR 4.59; 95% CI 3.99–5.27), thrombosis (ROR 1.36; 95% CI 1.04–1.78), and arterial occlusive disease (ROR 12.60; 95% CI 9.01–17.62) at the PT level, the clinical priority for each of these signals was weak. Nevertheless, coronary artery occlusion, cardiac failure congestive, cerebrovascular accident, and loss of consciousness were moderate clinical priority signals in our analysis. A disproportionality analysis by Woods et al. (28) reported that risankizumab was associated with a significantly cerebrovascular accident signal compared to all other drugs in the FAERS (ROR 2.48; 95% CI 2.14–2.88). A pooled analysis of 17 clinical trials with risankizumab showed that the rates of MACE were 0.2 and 0.3 events per 100 patient-years in the short- and long-term analysis sets, respectively, which were consistent with rates in the PSOLAR study (14, 36). However, the 52-week results from the KEEPsAKE 1 study of risankizumab also did not observe MACE, but four events of herpes zoster and one event of oropharyngeal candidiasis were noted (37). Naik (4) also stated that the association of IL-23 inhibitors with cardiovascular events remained controversial, further validating our clinical priority results.

A network meta-analysis comparing the safety and benefit–risk profile of biologics for moderate-to-severe plaque psoriasis demonstrated that the estimated rates of AEs leading to treatment discontinuation were the lowest for risankizumab (0.5%, 95% CI: 0.2%–1.3%), followed by tildrakizumab (1.0%, 95% CI: 0.2%–4.4%), guselkumab (1.5% 95% CI: 0.7%–3.1%), ustekinumab (1.6%, 95% CI: 0.7%–3.4%), etanercept (1.7%, 95% CI: 0.6%–4.8%), and adalimumab (1.7%, 95% CI: 0.8%–3.6%) in the short term, among which risankizumab and guselkumab also had favorable efficacy profiles (8). Similarly, in the long term, risankizumab was associated with significantly lower odds of any AEs compared with the anti-IL-17 agents (ixekizumab and secukinumab), adalimumab, and ustekinumab (8). In a similar safety assessment of risankizumab, 0.5% and 3.6% cases discontinued the medicine due to AEs in the part A1 and part B, respectively (38), while this rate was 1.2% due to ustekinumab’s AEs (39). These results are consistent with a network meta-analysis conducted by the British Association of Dermatologists that risankizumab had the best safety profile and the most favorable benefit–risk profile compared with other biologics (40). The findings could be explained by the different action mechanisms of anti-IL-17 and anti-IL-23. IL-17 plays a role in host defense against bacterial, fungal, viral, and parasitic infections, whereas IL-23 is mainly involved in host protection against bacterial and parasitic infections (41).

According to the properties of the reports in the FAERS (primary suspect), tuberculosis (TB) is a treatment-emergent AE caused by risankizumab, probably because of the increased risk of infection. Latent TB was found in the IMMvent, UltIMMa-1, and UltIMMa-2 trials (11, 42), which was consistent with our results in **Supplementary Table S3**, but none developed active TB during the long-term follow-up period with risankizumab. However, the 48 cases of TB with ROR 8.00 (6.01–10.64) in **Table 3** refer to multiple forms of TB. We cannot rule out the possibility of developing active TB based on a large database, but risankizumab is not recommended once a patient was diagnosed with active TB (43). Only four cases (4/91, TB-associated PTs) in our study received anti-TB prophylaxis (three isoniazid and one rifampin). The absence of anti-TB prophylaxis may be explained by the fact that latent TB is mostly disease-free and the probability of progression to active TB is 5%–15% (44). Risk factors for hepatotoxicity and drug interactions should be considered when selecting anti-TB therapy. The isoniazid for treatment of latent TB may discontinue risankizumab treatment because of increased liver injury (43). However, it is necessary to evaluate patients for TB infection and consider anti-TB therapy prior to initiating treatment with risankizumab and to monitor patients for signs and symptoms of TB during and after risankizumab treatment. All risankizumab-associated TB cases were from psoriatic patients, probably because risankizumab was not approved by the FDA for Crohn’s disease until June 2022. The data on Crohn’s disease are very limited, and whether there is a significant difference between psoriatic patients and who suffered from Crohn’s disease need more updated data in the FAERS.

In the long-term integrated safety study of risankizumab, there were no reports of lymphoma or hematological malignancy, whereas basal cell carcinoma and cutaneous squamous cell carcinoma were reported in 23 and 14 patients, respectively (14). In addition, 17 deaths were reported, of which 16 were treatment-emergent deaths, including sudden cardiac death, myocardial infarction, congestive heart failure, cardiac arrest, intestinal adenocarcinoma, pancreatitis, and hepatic cirrhosis. A phase 3 clinical trial reported one malignancy of risankizumab at 360 mg for moderate-to-severe active Crohn’s disease (31). Consistently, risankizumab-associated AEs resulted in more serious than non-serious outcomes (68.57% vs. 31.43%). Furthermore, statistical differences (*P* < 0.05) of these AEs were observed between reports with serious and non-serious outcomes (**Table 4**). We also found a significant signal for risankizumab at the SOC level of neoplasms benign, malignant, and unspecified (n = 1,391, ROR_025_ = 1.19) in the present study. Interestingly, in our data analysis, some unexpected and new significant AE signals of risankizumab, which were not reported in the drug label, such as cataract, pancreatitis, diabetes mellitus, stress, nephrolithiasis, cholelithiasis, and thrombosis, were also detected. The TTO and WSP analysis indicated that most of the AEs occurred within the first 3 months (moderate: 115 days; weak: 124 days) after risankizumab initiation, and all of the AE signals had early failure type characteristics, suggesting that the risk of risankizumab-related AE occurrence gradually decreased over time. Therefore, clinicians should be aware of these new, severe, and moderate clinical priority signals, and the FDA can modify the label and give warnings if necessary, especially as the use of risankizumab is becoming more widespread in patients with psoriasis and Crohn’s disease.

Notably, there were several limitations of our study, mainly related to the inherent nature of all pharmacovigilance databases. A causal relationship cannot be established between drug exposure and the occurrence of AEs because disproportionality analysis only provides an evaluation of the signal strength, which is only statistically significant. Due to the lack of a denominator and the under-reporting phenomenon, ROR and its magnitude do not quantify the true risk in clinical practice. Thus, the incidence rate cannot be calculated by spontaneous reports. Moreover, multiple unmeasured confounders that may affect AEs (e.g., potential drug–drug interactions, the adjustment of therapeutic regimens, and laboratory and instrumental tests) were not included in the data analysis. Further experimental and prospective clinical studies are still needed to confirm the results.

## 5 Conclusion

The current study performed a comprehensive assessment of the safety profiles of risankizumab based on the real-world large-sample pharmacovigilance database. Among 10,235 reports of risankizumab as the PS and 79 significant AEs that at least 30 cases related to risankizumab were identified. Common AEs in SOC levels, such as infections and infestations; respiratory, thoracic, and mediastinal disorders; musculoskeletal and connective tissue disorders and vascular disorders; and severe AEs, including pneumonia, cerebrovascular accident, myocardial infarction, cardiac disorder, and hepatic cirrhosis, should be highly concerned. Notably, among the 79 PTs, 37 PTs were classified as unexpected AEs, which were uncovered in the drug label. Moreover, 59 (74.68%), 20 (25.32%), and 0 PTs were graded as weak, moderate, and strong clinical priorities based on the clinical priority scores, respectively. Most of the AEs occurred within the first 3 months after risankizumab initiation, and the risk of risankizumab-related AEs occurrence gradually decreased over time. Our pharmacovigilance study may play a central role in facilitating the risk–benefit assessment of risankizumab with vigorous long-term surveillance, particularly for unexpected AEs.

## Data availability statement

The original contributions presented in the study are included in the article/**Supplementary Material**. Further inquiries can be directed to the corresponding author.

## Ethics statement

This study was approved (No. 20220185) by the institutional ethics board of the Union Hospital of Tongji Medical College of Huazhong University of Science and Technology.

## Author contributions

YS: conceptualization, methodology, data curation, software, formal analysis, and writing—review and editing. JC and YD: data curation, validation, and revision. QZ: conceptualization, methodology, data curation, software, formal analysis, funding acquisition, supervision, and writing—review and editing. All authors contributed to the article and approved the submitted version.
