# Supplementary material for: Adverse events with risankizumab in the real world: postmarketing pharmacovigilance assessment of the FDA adverse event reporting system
Source: Front Immunol. 2023 May 15;14:1169735. doi: 10.3389/fimmu.2023.1169735 (PMC10225532; doi:10.3389/fimmu.2023.1169735)
Supplement: Supplementary file 1 [file Table_1.docx]

**Supplementary Table S1**. Calculation of reporting odds ratio (ROR).

|  | Reports with target AE | Reports without target AE |
| --- | --- | --- |
| Reports with risankizumab | a | b |
| Reports without risankizumab | c | d |

a, number of reports containing both the target drug (risankizumab) and target AE; b, number of reports containing other AEs of the target drug; c, number of reports containing the target AE of other drugs; d, number of reports containing other drugs and other AEs.

AEs, Adverse Events; ROR, Reporting Odds Ratio; CI, confidence interval.

The calculation formulas are shown below:

1. ROR=ad/b/c
2. 95%CI=e^ln(ROR)±1.96(1/a+1/b+1/c+1/d)^0.5^

**Supplementary Table S2**. A rating scale assessing clinical priority of disproportionality signals.

| **Assessment items** | **2 points** | **1 point** | **0 point** |
| --- | --- | --- | --- |
| Number of target events | >50 | 10-50 | <10 |
| ROR | >5 | 2-5 | 1-2 |
| Mortality proportion | >50% | 25-50% | <25% |
| IMEs or DMEs | DME | IME | None |
| Relevant evidence evaluation | ++ | + | **-** |

Mortality proportion: percentage of cases in which death was reported as an outcome in the overall cases report for a particular adverse event. IMEs and DMEs are developed and updated by EMA (European Medicines Agency, 2022). ++ : AEs are mainly from the FDA Prescribing Information, the Summary of Product Characteristics of risankizumab posted by the MHRA, Phase 2/3 RCTs, or systematic reviews, with biological plausibility. + : AEs are mainly from other clinical trials, observational studies, or case reports/series with potential biological plausibility. - : AEs only emerging from disproportionality analyses.

AEs, Adverse Events; DMEs, Designated Medical Events; IMEs, Important Medical Events; MHRA, Medicine and Healthcare Products Regulatory Agency; RCTs, Randomized Controlled Trials; ROR, Reporting odds ratio.

**Supplementary Table S3. Signal strength of the Preferred Term (PT) and the clinical priority assessing results (n** < **30).**

| SOC | PT | Cases (n) | ROR  (95% two-sided CI) | Death (n) | IME/DME | Relevant evidence  evaluation | Priority level  (score) |
| --- | --- | --- | --- | --- | --- | --- | --- |
| Blood and lymphatic system disorders | Increased tendency to bruise | 10 | 2.57 (1.38-4.78) | 0 | NA | - | Weak (2) |
| Cardiac disorders | Pericardial effusion | 20 | 1.94 (1.25-3.01) | 0 | IME | - | Weak (2) |
|  | Cardiovascular disorder | 17 | 3.19 (1.98-5.13) | 1 | NA | - | Weak (2) |
|  | Coronary artery disease | 12 | 1.89 (1.07-3.32) | 0 | NA | - | Weak (1) |
| Ear and labyrinth disorders | Deafness | 28 | 2.12 (1.46-3.08) | 0 | DME | - | Weak (4) |
| Endocrine disorders | Thyroid disorder | 13 | 1.75 (1.01-3.01) | 0 | NA | - | Weak (1) |
| Eye disorders | Glaucoma | 18 | 1.95 (1.23-3.10) | 0 | IME | - | Weak (2) |
|  | Retinal detachment | 15 | 3.92 (2.36-6.51) | 0 | IME | - | Weak (3) |
|  | Macular degeneration | 13 | 2.35 (1.36-4.06) | 0 | IME | - | Weak (3) |
|  | Blindness unilateral | 13 | 2.17 (1.26-3.74) | 0 | IME | - | Weak (3) |
| Gastrointestinal disorders | Large intestine polyp | 11 | 2.92 (1.61-5.28) | 0 | NA | - | Weak (2) |
|  | Food poisoning | 10 | 3.95 (2.12-7.36) | 0 | NA | - | Weak (2) |
| General disorders and administration site conditions | Pre-existing condition improved | 14 | 4.34 (2.57-7.35) | 0 | NA | - | Weak (2) |
|  | Mass | 14 | 1.97 (1.17-3.34) | 0 | NA | - | Weak (1) |
|  | Vaccination site pain | 12 | 25.35 (14.21-45.21) | 0 | NA | - | Weak (3) |
| Hepatobiliary disorders | Hepatic steatosis | 21 | 2.66 (1.73-4.09) | 2 | NA | + | Weak (3) |
|  | Gallbladder disorder | 19 | 3.39 (2.16-5.32) | 0 | NA | - | Weak (2) |
| Infections and infestations | Skin infection | 26 | 4.44 (3.01-6.53) | 0 | NA | ++ | Weak (4) |
|  | Kidney infection | 24 | 2.28 (1.52-3.40) | 2 | IME | + | Weak (4) |
|  | Ear infection | 22 | 1.77 (1.16-2.69) | 0 | NA | + | Weak (2) |
|  | Latent tuberculosis | 21 | 17.29 (11.19-26.70) | 0 | NA | + | Weak (4) |
|  | Wound infection | 21 | 4.86 (3.16-7.46) | 0 | NA | ++ | Weak (4) |
|  | Abscess | 21 | 2.82 (1.84-4.33) | 0 | NA | - | Weak (2) |
|  | Bacterial infection | 20 | 2.39 (1.54-3.71) | 0 | NA | ++ | Weak (4) |
|  | Postoperative wound infection | 17 | 4.72 (2.93-7.61) | 1 | IME | + | Weak (4) |
|  | Appendicitis perforated | 16 | 16.97 (10.31-27.91) | 0 | IME | - | Weak (4) |
|  | Gastrointestinal infection | 15 | 3.53 (2.13-5.87) | 0 | NA | + | Weak (3) |
|  | Coronavirus infection | 14 | 1.88 (1.11-3.18) | 1 | NA | - | Weak (1) |
|  | Urosepsis | 13 | 3.00 (1.74-5.18) | 4 | IME | - | Weak (4) |
|  | Suspected COVID-19 | 13 | 2.89 (1.68-4.99) | 0 | NA | - | Weak (2) |
| Injury, poisoning and procedural complications | Head injury | 29 | 2.14 (1.48-3.08) | 1 | NA | - | Weak (2) |
|  | Foot fracture | 28 | 3.05 (2.1-4.42) | 0 | NA | - | Weak (2) |
|  | Rib fracture | 27 | 2.70 (1.85-3.95) | 1 | NA | - | Weak (2) |
|  | Wound | 27 | 1.72 (1.18-2.51) | 1 | NA | - | Weak (1) |
|  | Meniscus injury | 27 | 8.14 (5.56-11.91) | 0 | NA | - | Weak (3) |
|  | Skin laceration | 27 | 3.50 (2.39-5.11) | 0 | NA | + | Weak (3) |
|  | Lower limb fracture | 26 | 3.09 (2.10-4.55) | 0 | NA | - | Weak (2) |
|  | Spinal fracture | 26 | 2.59 (1.76-3.81) | 0 | IME | - | Weak (3) |
|  | Joint injury | 26 | 2.57 (1.74-3.77) | 0 | NA | - | Weak (2) |
|  | Scar | 23 | 2.78 (1.85-4.19) | 0 | NA | + | Weak (3) |
|  | Wrist fracture | 22 | 3.93 (2.58-5.97) | 0 | NA | - | Weak (2) |
|  | Tendon rupture | 20 | 4.45 (2.86-6.91) | 0 | NA | - | Weak (2) |
|  | Femur fracture | 17 | 1.64 (1.02-2.64) | 1 | IME | - | Weak (2) |
|  | Hand fracture | 17 | 4.80 (2.98-7.74) | 0 | NA | - | Weak (2) |
|  | Nerve injury | 16 | 2.87 (1.75-4.69) | 0 | NA | - | Weak (2) |
|  | Muscle rupture | 13 | 7.33 (4.24-12.67) | 0 | IME | - | Weak (4) |
|  | Joint dislocation | 13 | 2.77 (1.61-4.78) | 0 | NA | - | Weak (2) |
|  | Ligament sprain | 12 | 2.37 (1.34-4.18) | 0 | NA | - | Weak (2) |
|  | Paternal exposure during pregnancy | 11 | 19.62 (10.75-35.8) | 0 | NA | - | Weak (3) |
|  | Vaccination complication | 11 | 10.56 (5.82-19.19) | 0 | NA | - | Weak (3) |
|  | Arthropod bite | 11 | 3.39 (1.87-6.13) | 0 | NA | - | Weak (2) |
|  | Scratch | 11 | 2.37 (1.31-4.28) | 0 | NA | - | Weak (2) |
| Investigations | White blood cell count increased | 27 | 1.80 (1.23-2.63) | 1 | NA | + | Weak (2) |
|  | Mycobacterium tuberculosis complex test positive | 21 | 16.68 (10.8-25.75) | 0 | NA | + | Weak (4) |
|  | Blood glucose abnormal | 19 | 2.12 (1.35-3.32) | 0 | NA | - | Weak (2) |
|  | Blood sodium decreased | 16 | 2.01 (1.23-3.29) | 1 | NA | - | Weak (2) |
|  | Precancerous cells present | 12 | 16.65 (9.37-29.57) | 0 | NA | - | Weak (3) |
|  | Grip strength decreased | 10 | 2.47 (1.33-4.60) | 0 | NA | - | Weak (2) |
| Metabolism and nutrition disorders | Gout | 20 | 2.46 (1.58-3.81) | 1 | NA | - | Weak (2) |
| Musculoskeletal and connective tissue disorders | Rotator cuff syndrome | 28 | 5.34 (3.68-7.75) | 1 | NA | - | Weak (3) |
|  | Joint range of motion decreased | 26 | 4.69 (3.19-6.90) | 0 | NA | - | Weak (2) |
|  | Back disorder | 21 | 2.79 (1.81-4.28) | 0 | NA | - | Weak (2) |
|  | Exostosis | 19 | 5.74 (3.65-9.03) | 0 | NA | - | Weak (3) |
|  | Spinal stenosis | 16 | 4.12 (2.52-6.75) | 0 | NA | - | Weak (2) |
| Nervous system disorders | Nerve compression | 17 | 3.84 (2.38-6.19) | 0 | NA | - | Weak (2) |
| Renal and urinary disorders | Renal cyst | 10 | 2.46 (1.32-4.57) | 0 | NA | - | Weak (2) |
| Reproductive system and breast disorders | Ovarian cyst | 15 | 3.95 (2.38-6.57) | 0 | NA | - | Weak (2) |
|  | Prostatomegaly | 12 | 6.51 (3.69-11.51) | 0 | NA | - | Weak (3) |
| Respiratory, thoracic and mediastinal disorders | Pulmonary thrombosis | 29 | 4.67 (3.24-6.74) | 0 | IME | - | Weak (3) |
|  | Pulmonary fibrosis | 17 | 2.07 (1.28-3.33) | 2 | DME | - | Weak (4) |
|  | Pulmonary mass | 17 | 2.02 (1.25-3.25) | 1 | NA | - | Weak (2) |
|  | Sinus disorder | 17 | 1.80 (1.12-2.89) | 0 | NA | - | Weak (1) |
|  | Sinus congestion | 16 | 3.02 (1.84-4.93) | 0 | NA | - | Weak (2) |
|  | Pneumothorax | 15 | 1.95 (1.17-3.24) | 1 | IME | - | Weak (2) |
|  | Respiratory tract congestion | 13 | 2.16 (1.25-3.72) | 0 | NA | - | Weak (2) |
| Skin and subcutaneous tissue disorders | Rash macular | 29 | 1.57 (1.09-2.26) | 0 | NA | ++ | Weak (3) |
|  | Scab | 27 | 4.68 (3.20-6.84) | 0 | NA | - | Weak (2) |
|  | Skin ulcer | 27 | 2.16 (1.48-3.15) | 0 | NA | + | Weak (3) |
|  | Dermatitis exfoliative generalised | 22 | 8.27 (5.43-12.61) | 1 | DME | ++ | Moderate (7) |
|  | Skin haemorrhage | 16 | 2.27 (1.39-3.71) | 0 | NA | - | Weak (2) |
|  | Papule | 13 | 3.86 (2.24-6.66) | 0 | NA | - | Weak (2) |
| Vascular disorders | Poor peripheral circulation | 11 | 5.88 (3.25-10.66) | 0 | NA | - | Weak (3) |

ROR, reporting odds ratio; CI, confidence interval; IME, Important Medical Event; DME, Designated Medical Event.

NA, Not Applicable (for relevant criteria); n, number of cases.

**Supplementary Table S4. Differences in clinical characteristics of serious and non-serious reports (n < 30).**

| Types of AEs, n < 30 (%) | Serious cases | Non-serious cases | Statistic | *p* value |
| --- | --- | --- | --- | --- |
| Head injury | 25 (0.36) | 4 (0.12) | 4.198*^b^* | 0.04*^a^* |
| Pulmonary thrombosis | 23 (0.33) | 6 (0.19) | 1.557*^b^* | 0.212*^a^* |
| Rash macular | 23 (0.33) | 6 (0.19) | 1.557*^b^* | 0.212*^a^* |
| Deafness | 26 (0.37) | 2 (0.06) | 1.566*^b^* | 0.211*^a^* |
| Foot fracture | 27 (0.38) | 1 (0.03) | 10.112*^b^* | 0.001*^a^* |
| Rotator cuff syndrome | 26 (0.37) | 2 (0.06) | 1.566*^b^* | 0.211*^a^* |
| Meniscus injury | 24 (0.34) | 3 (0.09) | 5.187*^b^* | 0.023*^a^* |
| Rib fracture | 23 (0.33) | 4 (0.12) | 3.468*^b^* | 0.063*^a^* |
| Scab | 21 (0.30) | 6 (0.19) | 1.065*^b^* | 0.302*^a^* |
| Skin laceration | 20 (0.28) | 7 (0.22) | 0.381*^b^* | 0.537*^a^* |
| Skin ulcer | 21 (0.30) | 6 (0.19) | 1.065*^b^* | 0.302*^a^* |
| White blood cell count increased | 22 (0.31) | 5 (0.16) | 2.094*^b^* | 0.148*^a^* |
| Wound | 26 (0.37) | 1 (0.03) | 9.657*^b^* | 0.002*^a^* |
| Joint injury | 22 (0.31) | 4 (0.12) | 3.114*^b^* | 0.078*^a^* |
| Joint range of motion decreased | 19 (0.27) | 7 (0.22) | 0.246*^b^* | 0.620*^a^* |
| Lower limb fracture | 23 (0.33) | 3 (0.09) | 4.786*^b^* | 0.029*^a^* |
| Skin infection | 21 (0.30) | 5 (0.16) | 1.800*^b^* | 0.180*^a^* |
| Spinal fracture | 23 (0.33) | 3 (0.09) | 4.786*^b^* | 0.029*^a^* |
| Kidney infection | 17 (0.24) | 7 (0.22) | 0.057*^b^* | 0.811*^a^* |
| Scar | 18 (0.26) | 5 (0.16) | 1.005*^b^* | 0.316*^a^* |
| Dermatitis exfoliative generalised | 20 (0.28) | 2 (0.06) | 5.106*^b^* | 0.024*^a^* |
| Ear infection | 17 (0.24) | 5 (0.16) | 0.775*^b^* | 0.379*^a^* |
| Wrist fracture | 18 (0.26) | 4 (0.12) | 1.796*^b^* | 0.180*^a^* |
| Abscess | 18 (0.26) | 3 (0.09) | 2.870*^b^* | 0.090*^a^* |
| Back disorder | 16 (0.23) | 5 (0.16) | 0.567*^b^* | 0.451*^a^* |
| Hepatic steatosis | 18 (0.26) | 3 (0.09) | 2.870*^b^* | 0.090*^a^* |
| Latent tuberculosis | 17 (0.24) | 4 (0.12) | 1.497*^b^* | 0.221*^a^* |
| Mycobacterium tuberculosis complex test positive | 12 (0.17) | 9 (0.28) | 1.275*^b^* | 0.259*^a^* |
| Wound infection | 17 (0.24) | 4 (0.12) | 1.497*^b^* | 0.221*^a^* |
| Bacterial infection | 16 (0.23) | 4 (0.12) | 1.215*^b^* | 0.270*^a^* |
| Gout | 18 (0.26) | 2 (0.06) | 4.271*^b^* | 0.039*^a^* |
| Pericardial effusion | 16 (0.23) | 4 (0.12) | 1.215*^b^* | 0.270*^a^* |
| Tendon rupture | 19 (0.27) | 1 (0.03) | 6.496*^b^* | 0.011*^a^* |
| Blood glucose abnormal | 15 (0.21) | 4 (0.12) | 0.951*^b^* | 0.329*^a^* |
| Exostosis | 15 (0.21) | 4 (0.12) | 0.951*^b^* | 0.329*^a^* |
| Gallbladder disorder | 17 (0.24) | 2 (0.06) | 3.860*^b^* | 0.049*^a^* |
| Glaucoma | 16 (0.23) | 2 (0.06) | 3.455*^b^* | 0.063*^a^* |
| Cardiovascular disorder | 13 (0.19) | 4 (0.12) | 0.493*^b^* | 0.482*^a^* |
| Femur fracture | 15 (0.21) | 2 (0.06) | 3.056*^b^* | 0.080*^a^* |
| Hand fracture | 15 (0.21) | 2 (0.06) | 3.056*^b^* | 0.080*^a^* |
| Nerve compression | 16 (0.23) | 1 (0.03) | 5.157*^b^* | 0.023*^a^* |
| Postoperative wound infection | 16 (0.23) | 1 (0.03) | 5.157*^b^* | 0.023*^a^* |
| Pulmonary fibrosis | 17 (0.24) | 0(0.00) | 7.806*^b^* | 0.005*^a^* |
| Pulmonary mass | 17 (0.24) | 0 (0.00) | 7.806*^b^* | 0.005*^a^* |
| Sinus disorder | 9 (0.13) | 8 (0.25) | 1.930*^b^* | 0.165*^a^* |
| Appendicitis perforated | 12 (0.17) | 4 (0.12) | 0.308*^b^* | 0.579*^a^* |
| Blood sodium decreased | 12 (0.17) | 4 (0.12) | 0.308*^b^* | 0.579*^a^* |
| Nerve injury | 13 (0.19) | 3 (0.09) | 1.196*^b^* | 0.274*^a^* |
| Sinus congestion | 8 (0.11) | 8 (0.25) | 2.564*^b^* | 0.109*^a^* |
| Skin haemorrhage | 6 (0.09) | 10 (0.31) | 7.177*^b^* | 0.007*^a^* |
| Spinal stenosis | 14 (0.20) | 2 (0.06) | 2.665*^b^* | 0.103*^a^* |
| Gastrointestinal infection | 14 (0.20) | 1 (0.03) | - | 0.048*^c^* |
| Ovarian cyst | 12 (0.17) | 3 (0.09) | - | 0.416*^c^* |
| Pneumothorax | 14 (0.20) | 1 (0.03) | - | 0.048*^c^* |
| Retinal detachment | 14 (0.20) | 1 (0.03) | - | 0.048*^c^* |
| Coronavirus infection | 13 (0.19) | 1 (0.03) | - | 0.079*^c^* |
| Mass | 10 (0.14) | 4 (0.12) | - | 1.000*^c^* |
| Pre-existing condition improved | 7 (0.10) | 7 (0.22) | - | 0.153*^c^* |
| Blindness unilateral | 10 (0.14) | 3 (0.09) | - | 0.766*^c^* |
| Joint dislocation | 11 (0.16) | 2 (0.06) | - | 0.369*^c^* |
| Macular degeneration | 12 (0.17) | 1 (0.03) | - | 0.076*^c^* |
| Muscle rupture | 11 (0.16) | 2 (0.06) | - | 0.369*^c^* |
| Papule | 8 (0.11) | 5 (0.16) | - | 0.562*^c^* |
| Respiratory tract congestion | 8 (0.11) | 5 (0.16) | - | 0.562*^c^* |
| Suspected COVID-19 | 10 (0.14) | 3 (0.09) | - | 0.766*^c^* |
| Thyroid disorder | 13 (0.19) | 0 (0.00) | - | 0.013*^c^* |
| Urosepsis | 13 (0.19) | 0 (0.00) | - | 0.013*^c^* |
| Coronary artery disease | 11 (0.16) | 1 (0.03) | - | 0.119*^c^* |
| Ligament sprain | 11 (0.16) | 1 (0.03) | - | 0.119*^c^* |
| Precancerous cells present | 12 (0.17) | 0 (0.00) | - | 0.024*^c^* |
| Prostatomegaly | 9 (0.13) | 3 (0.09) | - | 0.764*^c^* |
| Vaccination site pain | 8 (0.11) | 4 (0.12) | - | 1.000*^c^* |
| Arthropod bite | 7 (0.10) | 4 (0.12) | - | 0.750*^c^* |
| Large intestine polyp | 8 (0.11) | 3 (0.09) | - | 1.000*^c^* |
| Paternal exposure during pregnancy | 11 (0.16) | 0 (0.00) | - | 0.022*^c^* |
| Poor peripheral circulation | 11 (0.16) | 0 (0.00) | - | 0.022*^c^* |
| Scratch | 4 (0.06) | 7 (0.22) | - | 0.044*^c^* |
| Vaccination complication | 6 (0.09) | 5 (0.16) | - | 0.338*^c^* |
| Food poisoning | 8 (0.11) | 2 (0.06) | - | 0.734*^c^* |
| Grip strength decreased | 5 (0.07) | 5 (0.16) | - | 0.304*^c^* |
| Increased tendency to bruise | 4 (0.06) | 6 (0.19) | - | 0.082*^c^* |
| Renal cyst | 8 (0.11) | 2 (0.06) | - | 0.734*^c^* |

The AEs listed above were AEs with significant signal strength (n < 30).

*^a^* Proportions were compared using Pearson χ^2^ test.

*^b^* The χ^2^ statistic of the Pearson chi-square test.

*^c^* Fisher’s exact test.

*p* value less than 0.05 were considered statistically significant.

**Supplementary Table S5. The results of time-to-onset analysis for signals in SOC level.**

| SOC | TTO (days) | | | Weibull distribution | | | | Failure type |
| --- | --- | --- | --- | --- | --- | --- | --- | --- |
|  | Cases |  | | Scale parameter | | Shape parameter | |  |
|  | n | Median (IQR) | Min-max | α | 95% CI | β | 95% CI |  |
| Blood and lymphatic system disorders | 75 | 180 (54.5-334.5) | 0-917 | 202.34 | 153.32-267.02 | 0.84 | 0.70-1.02 | Early failure |
| Cardiac disorders | 389 | 115 (16-313) | 0-997 | 130.07 | 106.50-158.85 | 0.51 | 0.47-0.56 | Early failure |
| Ear and labyrinth disorders | 38 | 29.5 (10-190) | 0-527 | 55.39 | 24.78-123.82 | 0.41 | 0.31-0.54 | Early failure |
| Endocrine disorders | 45 | 142 (18-343) | 0-794 | 135.66 | 68.48-268.72 | 0.44 | 0.34-0.57 | Early failure |
| Eye disorders | 111 | 79 (21-252) | 0-997 | 112.20 | 79.48-158.40 | 0.56 | 0.47-0.65 | Early failure |
| Gastrointestinal disorders | 393 | 100 (20-288) | 0-1043 | 123.34 | 100.78-150.94 | 0.50 | 0.46-0.55 | Early failure |
| General disorders and administration site conditions | 800 | 102 (14-281.25) | 0-1096 | 106.66 | 90.59-125.59 | 0.44 | 0.41-0.46 | Early failure |
| Hepatobiliary disorders | 90 | 177 (65.25-320.25) | 0-941 | 213.12 | 166.60-272.63 | 0.87 | 0.73-1.03 | Early failure |
| Immune system disorders | 383 | 91 (14-290) | 0-993 | 108.13 | 86.10-135.79 | 0.45 | 0.42-0.50 | Early failure |
| Infections and infestations | 586 | 112 (30-276.5) | 0-1065 | 145.60 | 128.09-165.50 | 0.65 | 0.61-0.70 | Early failure |
| Injury, poisoning and procedural complications | 543 | 95 (14-273.5) | 0-1096 | 102.47 | 84.00-125.01 | 0.44 | 0.40-0.47 | Early failure |
| Investigations | 291 | 125 (31-274.5) | 0-917 | 146.67 | 120.08-179.16 | 0.59 | 0.53-0.65 | Early failure |
| Metabolism and nutrition disorders | 145 | 188 (32-371) | 0-837 | 198.46 | 153.70-256.27 | 0.66 | 0.57-0.76 | Early failure |
| Musculoskeletal and connective tissue disorders | 505 | 119 (19-285) | 0-1082 | 132.69 | 112.61-156.35 | 0.55 | 0.51-0.59 | Early failure |
| Neoplasms benign, malignant and unspecified (incl cysts and polyps) | 349 | 193 (62-351) | 0-1058 | 218.32 | 189.92-250.98 | 0.78 | 0.71-0.85 | Early failure |
| Nervous system disorders | 478 | 78.5 (12-230.75) | 0-997 | 88.21 | 71.28-109.17 | 0.43 | 0.40-0.47 | Early failure |
| Pregnancy, puerperium and perinatal conditions | 16 | 141.5 (41-449.75) | 0-832 | 152.94 | 49.08-476.57 | 0.44 | 0.28-0.69 | Early failure |
| Product issues | 30 | 96.5 (18-328.75) | 0-1096 | 115.11 | 45.72-289.78 | 0.40 | 0.29-0.55 | Early failure |
| Psychiatric disorders | 175 | 107 (14-229) | 0-959 | 107.79 | 78.77-147.49 | 0.49 | 0.43-0.55 | Early failure |
| Renal and urinary disorders | 154 | 154.5 (43-300.25) | 0-1009 | 156.69 | 119.05-206.22 | 0.59 | 0.52-0.68 | Early failure |
| Reproductive system and breast disorders | 132 | 202 (34-351.25) | 0-1058 | 198.14 | 151.66-258.87 | 0.66 | 0.56-0.76 | Early failure |
| Respiratory, thoracic and mediastinal disorders | 540 | 108.5 (24.75-277.75) | 0-1059 | 134.27 | 114.81-157.04 | 0.56 | 0.52-0.60 | Early failure |
| Skin and subcutaneous tissue disorders | 687 | 78 (14-266) | 0-993 | 99.31 | 84.18-117.17 | 0.47 | 0.44-0.50 | Early failure |
| Social circumstances | 25 | 141 (65-318) | 0-669 | 178.79 | 102.82-310.89 | 0.73 | 0.52-1.02 | Early failure |
| Surgical and medical procedures | 326 | 177.5 (54.5-324.75) | 0-1009 | 213.04 | 185.55-244.59 | 0.81 | 0.74-0.89 | Early failure |
| Vascular disorders | 480 | 117.5 (17-295.75) | 0-1096 | 128.96 | 106.73-155.82 | 0.49 | 0.45-0.53 | Early failure |

n, number of cases with available time-to-onset; IQR, interquartile range; TTO, Time-to-onset. When TTO, is 0 days, the adverse event occurred within the same day with the therapy.
